# Insights into the molecular basis of the palmitoylation and depalmitoylation of NCX1

**DOI:** 10.1016/j.ceca.2021.102408

**Published:** 2021-07

**Authors:** Caglar Gök, Alice Main, Xing Gao, Zsombor Kerekes, Fiona Plain, Chien-Wen Kuo, Alan D. Robertson, Niall J. Fraser, William Fuller

**Affiliations:** a Institute of Cardiovascular & Medical Sciences, Sir James Black Building, University of Glasgow, Glasgow, G12 8QQ, United Kingdom; b School of Medicine, Ninewells Hospital, University of Dundee, Dundee, DD1 9SY, United Kingdom

**Keywords:** Palmitoylation, NCX1, Depalmitoylation, zDHHC-PATs, APT1, Thioesterase

## Abstract

Catalyzed by zDHHC-PAT enzymes and reversed by thioesterases, protein palmitoylation is the only post-translational modification recognized to regulate the sodium/calcium exchanger NCX1. NCX1 palmitoylation occurs at a single site at position 739 in its large regulatory intracellular loop. An amphipathic ɑ-helix between residues 740–756 is a critical for NCX1 palmitoylation. Given the rich background of the structural elements involving in NCX1 palmitoylation, the molecular basis of NCX1 palmitoylation is still relatively poorly understood. Here we found that (1) the identity of palmitoylation machinery of NCX1 controls its spatial organization within the cell, (2) the NCX1 amphipathic ɑ-helix directly interacts with zDHHC-PATs, (3) NCX1 is still palmitoylated when it is arrested in either Golgi or ER, indicating that NCX1 is a substrate for multiple zDHHC-PATs, (4) the thioesterase APT1 but not APT2 as a part of NCX1-depalmitoylation machinery governs subcellular organization of NCX1, (5) APT1 catalyzes NCX1 depalmitoylation in the Golgi but not in the ER. We also report that NCX2 and NCX3 are dually palmitoylated, with important implications for substrate recognition and enzyme catalysis by zDHHC-PATs. Our results could support new molecular or pharmacological strategies targeting the NCX1 palmitoylation and depalmitoylation machinery.

## Highlights

• The structural element that directs NCX1 palmitoylation interacts with zDHHC-PATs.
• NCX2 and NCX3 are dually palmitoylated.
• Arresting NCX1 within the Golgi or ER does not change its palmitoylation status.
• APT1 but not APT2 governs the subcellular organization of NCX1.
• APT1 catalyzes NCX1 depalmitoylation in the Golgi but not in the ER.

## Graphical abstract

**Figure:**
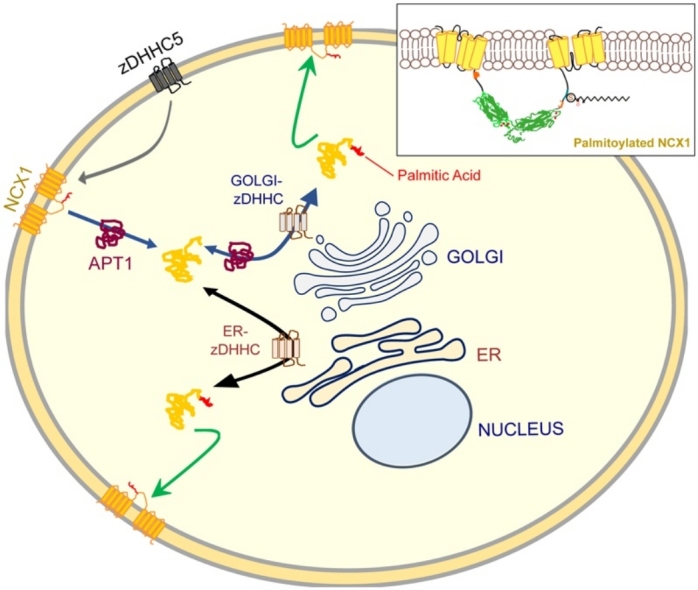

## 1 Introduction

The cardiac Na^+^/Ca^2+^ exchanger (NCX1) regulates cytosolic Ca^2+^ levels in myocytes and therefore cardiac contractility by controlling the bidirectional transport of Na^+^ and Ca^2+^ ions across the surface membrane. In healthy myocardium, NCX1 mainly generates an inward Na^+^ current and extrudes Ca^2^ (1 Ca^2+^: 3 Na^+^) [1,2]. Inappropriate NCX1 activity is associated with a number of severe cardiac pathologies including myocardial infarction [3], heart failure [4] and arrhythmias [5,6].

**Fig. 1:**
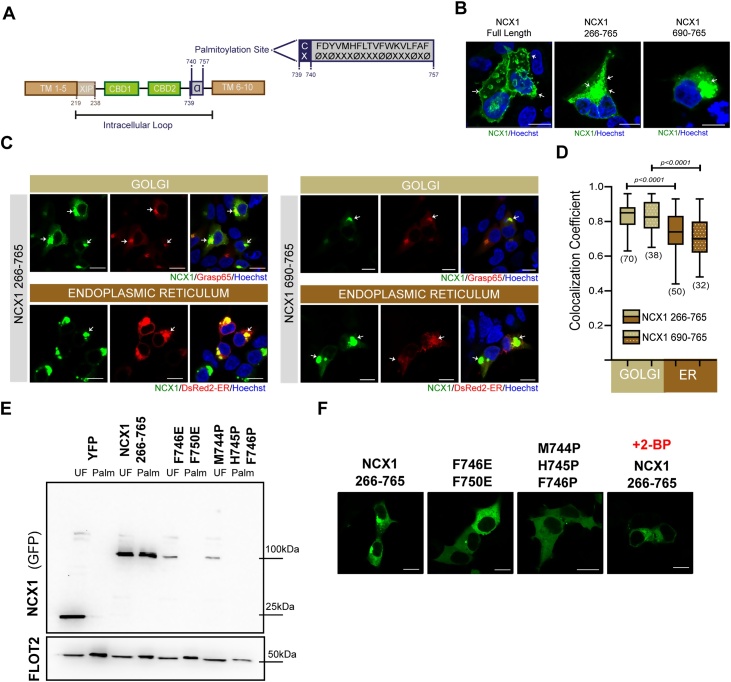
Tracking subcellular localization of NCX1 palmitoylation machinery. **(A)** Schematic of NCX1 structure equipped with 10 T M and a large intracellular loop accommodating Exchanger Inhibitory Peptide (XIP), Calcium Binding Domains (CBDs: CBD1 and CBD2) and palmitoylation site**, (B)** Example confocal images demonstrating localization of full length NCX1, NCX1^266–765^ and NCX1^690–765^ in HEK293 cells, **(C, D)** Both NCX1^266–765^ and NCX1^690–765^ colocalized with Golgi and ER (Pearson’s coefficient: 0.83 ± 0.0094 (SEM) for NCX1^266–765^/Golgi (n: 70) and 0.73 ± 0.017 (SEM) for NCX1^266–765^/ER (n: 50); 0.833 ± 0.013 (SEM) for NCX1^690–765^/Golgi (n: 38) and 0.70 ± 0.022 (SEM) for NCX1^690–765^/ER (n: 32)). Golgi localization of both NCX1 fragments were significantly greater than those localized in ER (unpaired *t*-test: p-value: <0.0001 for NCX1^266–765^/Golgi vs NCX1^266–765^/ER and NCX1^690–765^/Golgi vs NCX1^690–765^/ER, calculated by unpaired *t*-test) **(E, F)** Either breaking the ɑ-helix structure (M744 P/H745 P/F746 P) or introducing negatively charged aa in hydrophobic part of amphipathic helix (F746E/F750E) disrupted both palmitoylation and intracellular compartmentalization of NCX1. UF: unfractionated cell lysate; Palm: purified palmitoylated proteins. Scale bars: 5 μm.

Multiple regulatory mechanisms control NCX1. Two calcium binding domains (CBDs) in the large regulatory loop (Fig. 1A) facilitate allosteric regulation of NCX1 by intracellular calcium [7]. Calcium binding to CBD1 activates NCX1, while calcium binding to CBD2 opposes NCX1 inactivation [8,9]. The endogenous XIP domain, at the N terminal end of the same regulatory loop (Fig. 1A), inhibits NCX1 [10] but is usually sequestered by the phospholipid PIP2 [11]. Reversible palmitoylation is a key regulator of NCX1 activity [12,13] because it sensitizes NCX1 to XIP, and hence to PIP2, leading to enhanced NCX1-mediated transmembrane Ca flux when NCX1 is non-palmitoylated [14]. This effect of palmitoylation on NCX1 inactivation occurs without an apparent impact on the ability of calcium to activate NCX1 [15]. However, it is notable that the close proximity of CBD2 (502–690) to the XIP binding (709–720 [14]) and palmitoylation (C739) sites means that the conformational flexibility of the entire C terminal half of the NCX1 regulatory loop may change according to Ca occupancy of CBD2, whether XIP is sequestered by PIP2, and whether C739 is palmitoylated [7]. Hence understanding the cellular control of NCX1 palmitoylation is a high priority.

Palmitoylation relies on the direct conjugation of a fatty acid (usually 16C palmitic acid) onto cysteine residue(s) of a target protein via thioester bond [16]. This dynamic modification is catalyzed by a group of enzymes, called protein acyl-transferases (PATs), with a unique cysteine rich zinc-finger containing Asp-His-His-Cys (zDHHC) motif and reversed by depalmitoylating enzymes including acyl-protein thioesterases (APTs; APT1 and APT2) and lysosomal palmitoyl-protein thioesterases (PPTs; PPT1 and PPT2). NCX1 has one palmitoylated cysteine, which sits at the position 739 at the C terminal end of its large regulatory intracellular loop, close to the XIP binding site [14]. A single mutation from cysteine to alanine (C739A) at this position abolishes NCX1 palmitoylation [12,15,17,13]. An amphipathic ɑ-helix structure directly adjacent to the palmitoylated cysteine is required for palmitoylation of the exchanger [18,19]; however, how this particular structure, residing between residues 740 and 756, controls NCX1 palmitoylation remains unclear.

Given the structural insights into the underlying mechanism of NCX1 palmitoylation reported so far, our knowledge on enzymatic regulation of NCX1 palmitoylation is limited to the recent discovery of zDHHC5 and APT1 mediated palmitoylation and depalmitoylation of the exchanger [14]. zDHHC5 was found to interact with the exchanger and act as a sensor regulating the dynamic changes in NCX1 palmitoylation and therefore palmitoylation dependent structural re-arrangement within the NCX1 dimer. NCX1 palmitoylation in zDHHC5 KO cells was diminished, but not completely lost, which implies roles for multiple zDHHC-PATs in NCX1 palmitoylation [14].

Despite the considerable importance of palmitoylation in NCX1 physiology, there are still missing pieces regarding molecular specificity of NCX1 palmitoylation. In this study, we set out to investigate the spatial control of NCX1 palmitoylation, and how in turn this controls NCX1 localisation. We report that by interacting with the NCX1 palmitoylating enzymes, the amphipathic α-helix responsible for NCX1 palmitoylation directs the subcellular distribution of NCX1. By arresting NCX1 in intracellular compartments we determine that it is a substrate for both ER and Golgi-resident zDHHC-PATs. We also find that the same the amphipathic α-helix responsible for NCX1 palmitoylation directs dual palmitoylation of NCX2 and NCX3. Finally we report that ATP1 but not APT2 controls the spatial organisation of NCX1 in the cell, but that it is unable to depalmitoylate NCX1 in the ER. Our findings defining the molecular nature of NCX1 palmitoylation have important implications in the design of pharmacological tools to target NCX1 palmitoylation to cope with cardiac pathologies associated with abnormal exchanger activity.

## 2 Results

### 2.1 The palmitoylation machinery of NCX1 determines its subcellular localization

NCX1 architecture consists of 10 T M domains and a large intracellular loop positioned between TM5 and 6, which harbours 3 functionally key components: 20aa long XIP peptide, two CBDs and the palmitoylation site (Fig. 1A). Here, we first asked if we can track palmitoylated NCX1 in the cell via its palmitoylation site. Full length canine NCX1 tagged with YFP at position 266; NCX1^266Y^, [20] (left, Fig. 1B) and YFP- tagged NCX1 truncations corresponding to residues 266–765 (NCX1^266–765^) covering the CBDs and palmitoylation site (middle, Fig. 1B) and residues 690–765 (NCX1^690–765^) with only the palmitoylation site (right, Fig. 1B), were expressed in HEK293 cells. While NCX1 was situated at the cell surface when the TM domains are present in the structure, both truncations were populated in distinct intracellular puncta in the cells (Fig. 1B) which indicates that the palmitoylated state is retained in distinct compartment(s) in the absence of TM domains. We next hypothesized that NCX1 fragments containing the palmitoylation site are compartmentalized at intracellular organelles such as Golgi and Endoplasmic Reticulum (ER), where palmitoylating enzymes are abundant. We therefore co-expressed NCX1 fragments; either NCX1^266–765^ or NCX1^690–765^, with either Golgi (Grasp65-mcherry) or ER (DsER-red) markers (Fig. 1C), then analysed and quantified the degree of co-localization (Fig. 1D). Both fragments; NCX1^266–765^ and NCX1^690–765^, displayed a notable degree of spatial overlap with Golgi and ER markers. However the degree of co-localization with the Golgi marker was significantly greater than the ER marker for both NCX1 fragments (p-value: <0.0001 for NCX1^266–765^/Golgi vs NCX1^266–765^/ER and NCX1^690–765^/Golgi vs NCX1^690–765^/ER, calculated by unpaired *t*-test). In separate experiments we obtained similar results when we examined colocalization of NCX1^266–765^ with HA-tagged Golgin-84 (Golgi marker) or HA-tagged Ii (ER marker) (Supplementary Fig. 1).

Data herein support the notion that the subcellular organization of the NCX1 intracellular loop is controlled by its palmitoylation machinery. To prove the concept of “palmitoylation machinery” dependent subcellular distribution of NCX1, we investigated how NCX1^266–765^ localization is altered when its palmitoylation is drastically abolished by either (1) breaking the amphipathic ɑ-helix that is required for NCX1 palmitoylation (M744 P/H745 P/F746 P) or (2) introducing negatively charged aa; Glu- (E), to position 746 and 750 in hydrophobic part of the ɑ-helix (F746E/F750E) (Fig. 1E), or (3) incubating with a broad palmitoylating enzyme inhibitor, 2-Bromopalmitate (2-BP) [18,14,19]. NCX1 is no longer palmitoylated when either M744 P/H745 P/F746 P or F746E/F750E mutations are introduced (Fig. 1E). All experimental strategies aiming to prevent the formation of palmitoylation complex of NCX1 disrupted its organelle-specific retention (Fig. 1F).

### 2.2 The amphipathic ɑ-helix of NCX1 interacts with zDHHC-PATs

**Fig. 2:**
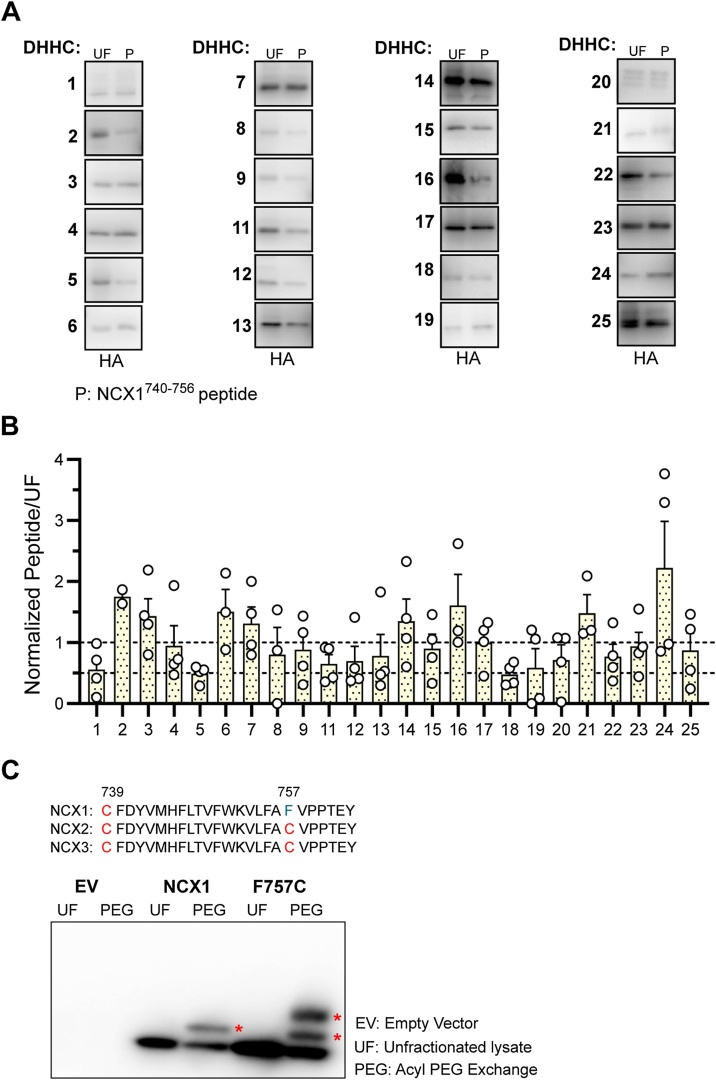
NCX1 interacts with zDHHC-PATs through its amphipathic ɑ-helix. **(A)** Example blots of zDHHC-PATs purified by custom made peptide corresponding to ɑ-helix structure of NCX1 (NCX1^740–756^) **(B)** All DHHC isoforms were pulled down with different propensities by the NCX1 peptide. **(C)** Sequence comparison of NCX1, NCX2 and NCX3. Acyl PEG exchange indicates single palmitoylation of NCX1 but dual palmitoylation of NCX2 and NCX3. EV: empty vector transfected cells; UF: unfractionated cell lysate; PEG: acyl PEG exchange.

Given the detailed knowledge regarding the structural organization of the NCX1 palmitoylation site, how NCX1 gets palmitoylated still remains unclear. The amphipathic ɑ-helix that is next to palmitoylated cysteine (C739) was previously identified as the “control unit” of NCX1 palmitoylation [18,19]. We asked if this ɑ-helix, corresponding to 740–756aa residues of NCX1 intracellular loop, is the structural component that interacts with palmitoylating zDHHC-PAT enzymes. Therefore, we transfected HEK293 cells with HA tagged zDHHC isoforms (24 isoforms), then purified the protein acyltransferases that are associated with NCX1 using a biotinylated custom-made ɑ-helix (NCX1^740–756^) peptide from cell lysates. Strikingly, we pulled down all zDHHC isoforms with different propensities to bind to the ɑ-helix peptide (Fig. 2A). It is thus conceivable that amphipathic helix may be recognized by a specific sequence/motif that can be found in all DHHC isoforms. zDHHC5 is the only palmitoylating enzyme reported to interact with NCX1 to date [14]. Apart from zDHHC5, several Golgi- (zDHHC3, zDHHC4, zDHHC7, zDHHC8, zDHHC15, zDHHC17 and zDHHC18), ER- (zDHHC1, zDHHC11, zDHHC13, zDHHC14, zDHHC16 and zDHHC19) and Golgi/ER- (zDHHC2, zDHHC9, zDHHC12 and zDHHC22) located protein acyl-transferases [[21], [22], [23], [24], [25], [26], [27]] were highly associated with ɑ-helix peptide (Fig. 2B). This strongly supports our findings that formation of the NCX1-palmitoylation complex requires the association between the amphipathic helix of the exchanger and Golgi, ER or Golgi/ER resident palmitoylating enzymes.

### 2.3 NCX2 and NCX3 are dually palmitoylated

Given our finding that the palmitoylation-directing NCX1 helix interacts with the zDHHC-PAT(s), we turned our attention to the same region of NCX2 and NCX3. These transporters both possess a cysteine in the equivalent position to C739, followed by the identical amphipathic α helix with a second cysteine at its C terminal end (position F757 in NCX1, Fig. 2C). We used a PEGylation assay, which exchanges palmitates for a 5 kDa PEG molecule, to measure the palmitoylation stoichiometry of F757C NCX1 and found it to be dually palmitoylated. Hence the zDHHC-PATs that recognise this structural element are capable of palmitoylating cysteines at either end of the helix.

### 2.4 NCX1 is palmitoylated even when it is arrested in either Golgi or ER

**Fig. 3:**
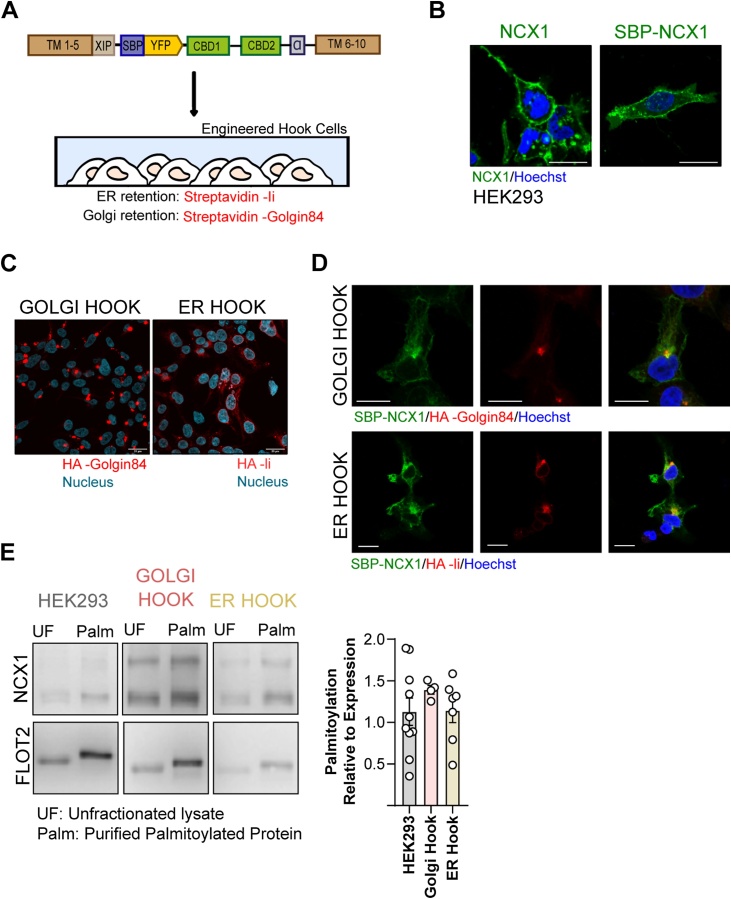
Establishing RUSH system to identify organelle specific NCX1 palmitoylation machinery. **(A)** Schematic illustrating the design of NCX1-RUSH system **(B)** SBP fusion to NCX1 did not alter the cellular distribution of NCX1. Scale bar: **1**0μm **(C)** Immunostained confocal images of HA-tagged hooks in Golgi-Hook and ER-Hook cell lines. Scale bar: 20 μm **(D)** Confocal imaging confirms that SBP-NCX1 (green) is retained in the appropriate cellular compartment when it is expressed in cells expressing Golgi or ER hooks. Scale bar: 10 μm **(E)** NCX1 is palmitoylated when it is retained in either Golgi or ER by RUSH (Palmitoylation level of NCX1 relative to its expression: 1.12 ± 0.05 (SEM) in HEK293 cells (n:10), 1.39 ± 0.02 (SEM) in Golgi Hook cells (n: 4) and 1.14 ± 0.05 (SEM) in ER Hook cells (n: 7); Unpaired t-test: HEK293 vs Golgi Hook p-value: 0.34; HEK293 vs ER Hook p-value:0.95; Golgi Hook vs ER Hook p-value: 0.23).

Based on our data presented here which dissected the interacting zDHHC-PATs of NCX1, we hypothesized that NCX1 can be palmitoylated in both the Golgi and the ER. To test our hypothesis, we employed Retention Using Selective Hook (RUSH) System [28]. RUSH system was originally introduced to analyse and quantify the trafficking of various proteins in live cells. This system is, in principle, a two-state assay which relies on the interaction between the hook, stably expressed in the intracellular compartment of interest, and Streptavidin Binding Protein (SBP) fused- reporter (Fig. 3A). Herein we adapted and re-purposed RUSH to further our understanding of the relationship between NCX1 and its palmitoylating enzymes. We generated two critical components of RUSH system: (1) SBP fused- full length canine NCX1 tagged with YFP at position 266 (NCX1^266Y^) (Fig. 3B), and (2) cell lines stably expressing either Golgi (Streptavidin-HA-Golgin84) or ER (Streptavidin-HA-Ii) hooks. We tested if SBP insertion into NCX1 causes any changes in subcellular localization of NCX1 and we found that fusing SBP to NCX1 had no effect on cellular distribution of (full length) NCX1 in HEK293 cell; localized through plasma membrane as expected (Fig. 3B). HA-tagged hook proteins displayed classic ER and Golgi localisation (Fig. 3C). When SBP-NCX1 was expressed in either Golgi- or ER- hook cells, it was predominantly observed in the same compartment as the hook (Fig. 3D). These validate the reliability and the suitability of our RUSH system for the aim of this study. Finally we measured the palmitoylation level of NCX1 in both Golgi- and ER-hook cells to check if NCX1 is palmitoylated when it is restricted in either cellular compartment (Fig. 3D). We found that NCX1 was equally palmitoylated regardless of whether its expression was limited to either intracellular compartment.

### 2.5 Depalmitoylation machinery of NCX1

**Fig. 4:**
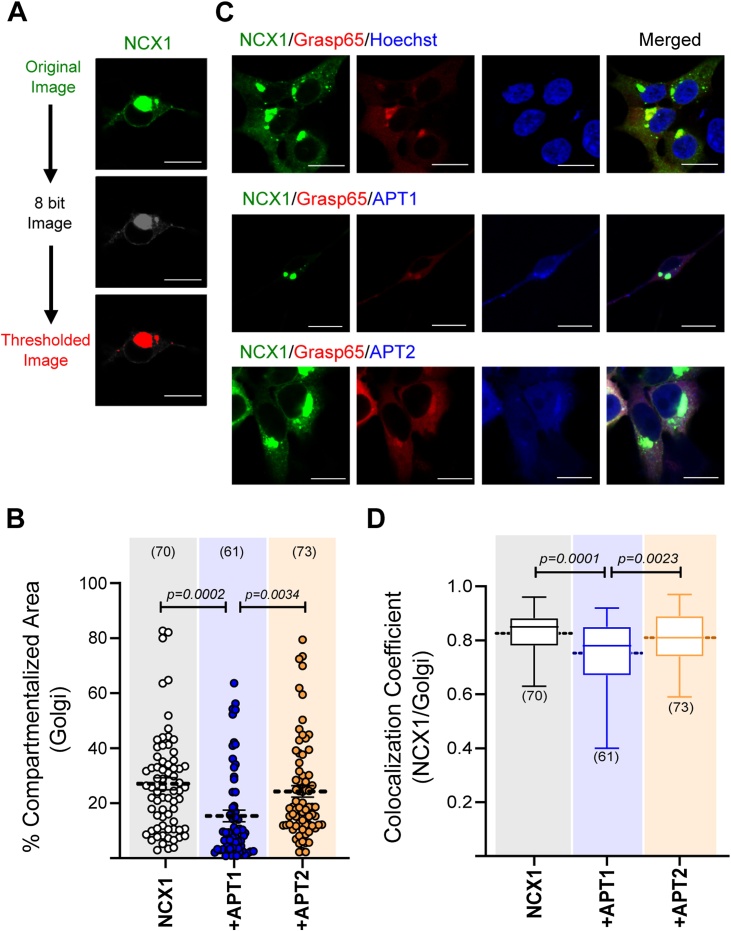
The activity of APT1 but not APT2 diminished the amount of palmitoylated NCX1 in Golgi and colocalization of NCX1/Golgi. **(A)** Steps for image analysis to quantify the size of “compartmentalized NCX1″ in the cell, Scale bar:10 μm **(B)** APT1 but not APT2 altered the size of NCX1 puncta in the Golgi (Golgi: 27.03 % ±1.86 (SEM) for NCX1^266–765^ only (n: 70), 15.57 % ±1.96 (SEM) for NCX1^266–765^/APT1 (n: 61), 24.26 % ±2.09 (SEM) for NCX1^266–765^/APT2 (n: 73); unpaired *t*-test: NCX1^266–765^ only vs NCX1^266–765^/APT1 p-value: 0.0003 and NCX1^266–765^ only vs NCX1^266–765^/APT2 p-value: 0.363). **(C, D)** Golgi colocalization of NCX1 was diminished when APT1 but not APT2 was expressed (Pearson’s coefficient: 0.83 ± 0.0094 (SEM) for NCX1^266–765^ only/Golgi (n: 70); 0.75 ± 0.015 (SEM) for NCX1^266–765^ +APT1/Golgi (n: 61) and 0.81 ± 0.01 (SEM) for NCX1^266–765^ +APT2/Golgi (n: 73); p-values: 0.0001 for NCX1^266–765^ only vs NCX1^266–765^ +APT1; 0.0023 for NCX1^266–765^ +APT1 vs NCX1^266–765^ +APT2 and 0.17 for NCX1^266–765^ only vs NCX1^266–765^ +APT2, calculated by unpaired *t*-test). Scale bar:10 μm.

We recently identified that APT1 but not APT2 interacts with NCX1 by LC–MS/MS following peptide purification from heart and brain tissues [14]. Furthermore, pharmacologically impeding APT1 activity in cells increased palmitoylated NCX1 levels and FRET signals between NCX1 pairs. Here, we tested if we can probe the activity of depalmitoylation machinery of NCX1 in the cell using its palmitoylation site. A YFP tagged NCX1 fragment covering palmitoylation site; NCX1^266–765^, was over-expressed in HEK293 cells together with either APT1- or APT2- tagged with CFP, or alone as a control. In order to detect the activity of depalmitoylation machinery of NCX1, we first calculated the size of the compartmentalized NCX1 population in the cell in proportion to the total cell size for each cell (Fig. 4A). Using fluorescent markers of the Golgi and ER, we calculated the area of palmitoylated NCX1 in the Golgi or ER separately, in the presence and absence of APT1 or APT2 overexpression. The size of the Golgi-localized puncta containing the palmitoylated NCX1 (fragment) was significantly smaller in the cells over-expressing APT1 than control group. In contrast, over-expressing APT2 did not alter the size of populated NCX1 in Golgi in comparison to control group (Fig. 4B). This implies that over-expression of APT1 promoted NCX1 depalmitoylation, therefore reduced the amount of palmitoylated NCX1 which is compartmentalized in the Golgi. To validate this, we next assessed Golgi co-localization of NCX1 after either APT1 or APT2 over-expression (Fig. 4C, D). HEK293 cells were transfected with NCX1^266–765^ alone or together with either APT1 or APT2. Over-expression of APT1 but not APT2 caused less Golgi co-localization of NCX1 than the control group. We conclude that the enhanced NCX1 depalmitoylation caused by APT1 over-expression in these cells diminished the amount of palmitoylated NCX1 localized in the Golgi.

**Fig. 5:**
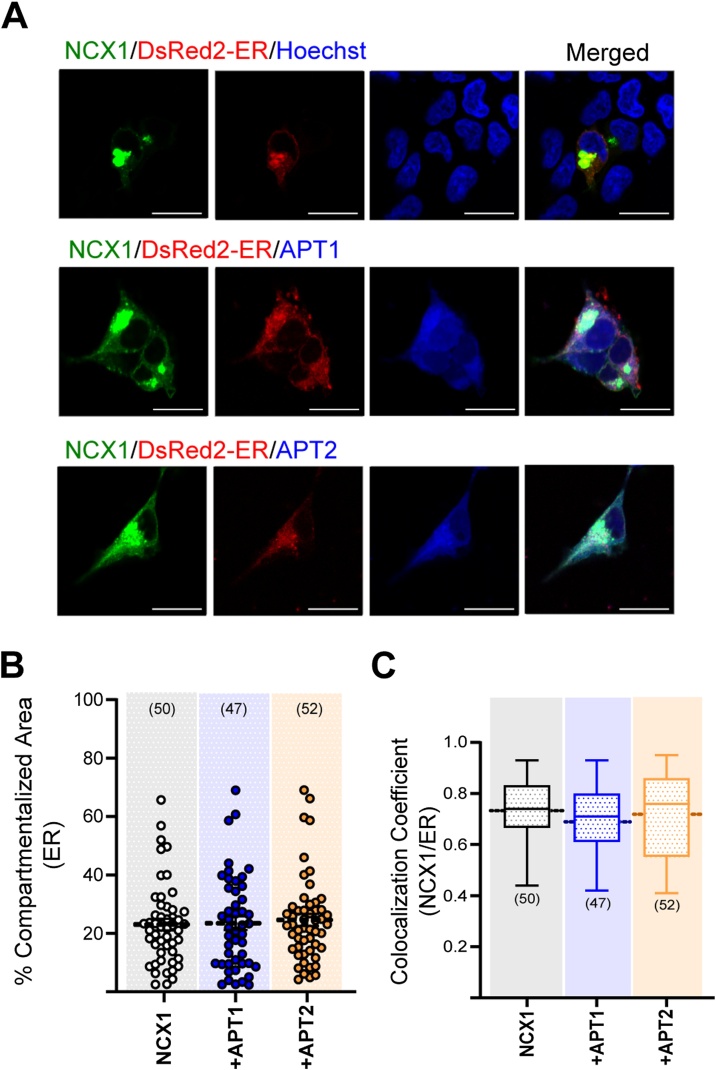
APT1 and APT2 did not affect the size of compartmentalized NCX1 in ER and colocalization of NCX1/ER. **(A)** Confocal images of HEK293 cells overexpressing NCX1/ER, NCX1/ER/APT1 and NCX1/ER/APT2, Scale bar: 10 μm **(B, C)** Neither APT1 nor APT2 affected the size of the NCX1 compartmentalized in ER (ER: 21.97 % ±1.766 (SEM) for NCX1^266–765^ only (n: 50), 21.8 % ±2.134 (SEM) for NCX1^266–765^/APT1 (n: 47), 22.08 % ±1.599 (SEM) for NCX1^266–765^/APT2 (n: 52); p-values: 0.881 for NCX1^266–765^ only vs NCX1^266–765^ +APT1; 0.583 for NCX1^266–765^ +APT1 vs NCX1^266–765^ +APT2 and 0.723 for NCX1^266–765^ only vs NCX1^266–765^ +APT2, calculated by unpaired *t*-test) and NCX1^266–765^/ER colocalization (Pearson’s coefficient: 0.73 ± 0.018 (SEM) for NCX1^266–765^ only/ER (n: 50); 0.69 ± 0.019 (SEM) for NCX1^266–765^ +APT1/ER (n: 47) and 0.71 ± 0.023 (SEM) for NCX1^266–765^ +APT2/ER (n: 52); p-values: 0.119 for NCX1^266–765^ only vs NCX1^266–765^ +APT1; 0.474 for NCX1^266–765^ +APT1 vs NCX1^266–765^ +APT2 and 0.489 for NCX1^266–765^ only vs NCX1^266–765^ +APT2, calculated by unpaired *t*-test).

Next we considered the population of palmitoylated NCX1 fragment found localized in the ER. Overexpression of neither APT1 nor APT2 altered the size of NCX1 puncta in this compartment (Fig. 5A, B). Nor did expression of APT1 or APT2 alter the colocalization of NCX1 with an ER marker (Fig. 5A, C). This suggests that APT1 only depalmitoylates NCX1 when it is in the Golgi.

## 3 Discussion

Palmitoylation is a critical regulator of NCX1 function [14], yet relatively little is known about the cellular mechanisms controlling NCX1 palmitoylation. Detailed mutation screening around the palmitoylation site has earlier described two crucial components of NCX1 palmitoylation: (1) the palmitoylated Cys- at position 739 [15], and (2) an amphipathic helix; a secondary structure element sitting between position 740 and 756 [18,19]. Here, we uncover the molecular identity of the NCX1 palmitoylation machinery using an intersectional approach. We also demonstrate that the palmitoylation-depalmitoylation cycle controls the spatial organization of NCX1 in the cell.

### 3.1 Spatial control of NCX1 palmitoylation

We first noted that NCX1 fragments (either NCX1^266–765^ or NCX1^690–765^) covering the palmitoylation site are compartmentalized in both Golgi and ER. Our previous investigations identified the Golgi as the main cellular location in which NCX1^266–765^ became localised [15,18,19], in keeping with the role of the Golgi as the cellular palmitoylation ‘hub’ [29,30]. In the present study, we confirmed the greatest quantitative overlap of palmitoylated NCX1 is with a Golgi marker, but that a significant proportion of palmitoylated NCX1 is also in the ER. This implies that the NCX1 palmitoylation site is recognised by both Golgi and ER- located zDHHC-PATs and that the NCX1 fragment gets anchored to the membrane following its palmitoylation. Supporting this, abolishing NCX1 palmitoylation by disrupting the palmitoylation machinery of the exchanger; either using broad inhibitor of palmitoylating enzymes; 2-BP, or breaking the ɑ-helix structure by M744 P/H745 P/F746 P or F746E/F750E, prevented this particular distribution of NCX1 in these compartments. Secondly, we found a peptide representing the NCX1 amphipathic helix interacts physically with Golgi, ER and Golgi/ER located palmitoylating enzymes. Third, with the help of RUSH system, we detected NCX1 to be equally palmitoylated when it is confined in either Golgi or ER. *What do these observations tell us?* The important take-home message here is that substrate recognition by zDHHC-PATs occurs at the ɑ-helix structure within the NCX1 intracellular loop, and NCX1 can be palmitoylated locally in either Golgi or ER which is determined by the nature of palmitoylation complex. The notion of local palmitoylation has been previously hypothesised for several proteins [31]. We suggest that integral membrane proteins like NCX1 can be palmitoylated in multiple compartments by multiple zDHHC-PATs.

It is well-established by several studies that palmitoylating enzymes have specific substrate preferences [[32], [33], [34], [35], [36]]. In our experiments, we purified numerous zDHHC-PATs with different efficiencies using a biotinylated peptide based on the NCX1 amphipathic ɑ-helix. We speculate that this ɑ-helix might be recognized by a motif common to multiple zDHHC-PATs, namely the active site. The binding of the zDHHC-PATs to this ɑ-helix peptide with different propensities might be due to the difference in coverage of this specific “*ɑ-helix binding motif*” in these acyltransferases.

We recently showed that zDHHC5 interacts with and palmitoylates NCX1 and alters NCX1-NCX1 FRET as a result of local changes around palmitoylation site within the intracellular loop induced by zDHHC5 mediated palmitoylation [14]. However, we also reported that NCX1 was still palmitoylated in zDHHC5 KO cells, implying that other zDHHC-PATs might involve in NCX1 palmitoylation. The data presented here clarify that NCX1 is a substrate for multiple zDHHC-PATs, not only for zDHHC5.

### 3.2 Enzymatic control of NCX1 palmitoylation

Our finding that NCX2 and NCX3 are dually palmitoylated at the N and C termini of the amphipathic α-helix has important implications for both substrate recognition and enzyme catalysis by zDHHC-PATs. The fact that the same amphipathic helix can direct palmitoylation of cysteines that are 18 residues apart from each other implies that proximity but not absolute position relative to this helix governs cysteine palmitoylation. This is important because it implies that algorithms that attempt to score palmitoylation sites based on their position relative to particular amino acids cannot be used to identify palmitoylation sites in the same way that they have been successfully used to identify phosphorylation sites.

With respect to catalysis, the ‘lock and key’ model of enzyme catalysis envisions a scenario in which the substrate ‘key’ fits the active site ‘lock’ in a single conformation. For the same recognition element to promote palmitoylation of amino acids so far apart in three dimensional space in NCX2 and NCX3 implies the substrate must be presented to the active site in a manner that can support palmitoylation in two positions (or alternatively that different zDHHC-PATs palmitoylate the cysteines at each end of the helix). Again, this contrasts with the behaviour of protein kinases, which is generally much more strict. Evidently the crystal structure of the zDHHC-PATs has greatly increased our understanding of this class of enzymes [37], but a co-crystal structure of a zDHHC-PAT with a substrate is a high priority.

Our finding that NCX1 is palmitoylated immediately after translation implies that this regulatory mechanism controls NCX1 activity from very early in the lifetime of the protein. Since palmitoylation controls XIP sensitivity of NCX1, our results suggest that NCX1 is sensitive to XIP throughout its lifetime in the cell. Ultimately the toxicity of NCX1 mediated cellular Ca overload means understanding the NCX1 regulatory mechanisms is a high priority. The complex interplay between NCX1 palmitoylation and the ionic (Na, Ca, pH) and phospholipid (PIP2) regulatory mechanisms evidently merits further investigation. For example, Ca occupancy of CBD2 opposes NCX1 inactivation [8]. The fact that palmitoylation promotes XIP binding close to CBD2 [14] and enhances the ability of NCX1 to inactivate [15] suggests functional interplay between CBD2 and the palmitoylation site may occur. The mechanism underlying Na-dependent inactivation remains enigmatic, but ultimately involves XIP engaging its binding site [10], so is likely regulated by palmitoylation. On the other hand, inhibition of NCX1 by reduced intracellular pH involves regions of the exchanger distant from the palmitoylation site [38], implying the two regulatory mechanisms are likely independent of each other. Leveraging the ability of NCX1 palmitoylation to protect cells from toxic Ca overload by promoting both inactivation and internalisation of NCX1 [15] may hold therapeutic promise. Our findings indicate that multiple zDHHC-PATs could be targeted to achieve such a goal. The central importance of the NCX1 amphipathic helix in recruiting both palmitoylating and depalmitoylating machinery will make this goal challenging.

### 3.3 Spatial control of NCX1 depalmitoylation

Data presented in this investigation shows that APT1 but not APT2 controls subcellular distribution of NCX1. Curiously, although APT1 is clearly capable of depalmitoylating NCX1, ER-localized palmitoylated NCX1 is unavailable to APT1. We suggest that APT1 is excluded from the ER, and that its ability to depalmitoylate NCX1 (and likely other substrates) hinges on their proximity in the same membrane compartment, rather than simple diffusion. This contrasts with the suggestion that the cytosolic pool of APT1 is responsible for substrate depalmitoylation in all membrane compartments [39]. Since APT1 is membrane-anchored by palmitoylation near its amino terminus [40], our findings imply that (unlike Golgi and plasma membrane zDHHC-PATs) ER-localized zDHHC-PATs cannot palmitoylate APT1 to recruit it into the ER. Whether other thioesterases depalmitoylate ER-localized substrates remains to be determined. When full-length NCX1 is trapped in the ER its palmitoylation level matches the level observed in other cellular compartments, despite the fact that it is unavailable to APT1 in the ER. Hence, we do not rule out the possibility that NCX1 is a substrate for other depalmitoylating enzymes. Overall, our data suggest that the switch mechanism between NCX1- palmitoylation and depalmitoylation governs the spatial organization of NCX1 in the cell; palmitoylation-depalmitoylation cycle could tune the shuttle of the exchanger between plasma membrane and “palmitoylating” compartments.

### 3.4 Limitations

Although experimental conditions were optimised to minimise non-specific binding, we acknowledge that overexpressed integral membrane proteins such as zDHHC-PATs can be susceptible to non-specific affinity purification. As such, we cannot rule out the possibility that some zDHHC-PATs co-purify non-specifically with our NCX1 peptide. Notwithstanding this point, our functional and imaging experiments clearly support the concept that NCX1 is palmitoylated by multiple zDHHC-PATs in multiple cellular compartments.

In this investigation we focussed entirely on NCX1 palmitoylation and depalmitoylation mechanisms in HEK cells. The overlap between the repertoire of zDHHC-PATs expressed in cardiac muscle is substantial [41,42]. We have already established that APT1 but not APT2 mediates NCX1 depalmitoylation in both HEK cells and cardiac muscle [14]. While we cannot rule different cellular mechanisms controlling NCX1 palmitoylation in cardiac muscle, we suggest the principals governing NCX1 palmitoylation identified in this study are broadly applicable to other cell types.

### 3.5 Conclusion

Palmitoylation is a critical process for physiological regulation of NCX1. Here we bring an important aspect into NCX1 palmitoylation by defining its molecular identity. Results of this study could possibly pave the way for new molecular/pharmacological strategies targeting the NCX1 palmitoylation/depalmitoylation machinery. As we now know that zDHHC-PATs and APT1 physically interact with NCX1 at amphipathic ɑ-helix, manipulating the availability of this region for binding of palmitoylating and depalmitoylating enzyme would be helpful to tune NCX1 physiology. More broadly, this investigation specifically supports the concept of ‘local palmitoylation’. Several zDHHC-PATs localised in different subcellular compartments are clearly capable of palmitoylating NCX1. We suggest that a similar principal will apply to numerous other palmitoylated integral membrane proteins as they move through the secretory pathway.

## 4 Methods

### 4.1 Cell lines, Plasmids and transfection

HEK293 cells and HEK293 derived FT293 cells stably expressing Golgi- (Golgin84), ER- (Ii) hook were generated using Flp-In T-Rex System (Invitrogen). Plasmids expressing YFP tagged full length NCX1 [20], YFP tagged NCX1 fragments covering the regions of NCX1 intracellular loop and YFP tagged NCX1 with M744 P/H745 P/F746 P or F746E/F750E mutations [18,19] were described previously. Golgi marker; mcherry tagged Grasp65, and ER marker; DsER-red, were used to identified co-localization with these organelles [15]. CFP tagged- APT1 and APT2 plasmids were a kind gift from Prof Michael J. Shipston (University of Edinburgh). Transfection of plasmid constructs was achieved using Lipofectamin 2000 (Invitrogen) according to manufacturer’s instructions.

### 4.2 Palmitoylation assays

Resin-assisted capture of acylated proteins (Acyl-RAC) was performed to purify palmitoylated proteins as described in detail elsewhere [15,18,19]. Free cysteine residues were first blocked with MMTS, then thioester bonds were cleaved with neutral hydroxylamine (HA). Palmitoylation level of NCX1 is calculated and presented as the amount of palmitoylated NCX1 purified relative to its abundance in the corresponding unfractionated cell lysate.

To measure palmitoylation site occupancy we used a PEGylation assay that exchanges palmitates for a 5 kDa PEG molecule [43]. Briefly, free cysteines were alkylated with maleimide, then previously palmitoylated cysteines revealed with neutral hydroxylamine and PEGylated with 5 kDa methoxypolyethylene glycol maleimide.

### 4.3 Peptide pull-down assay

HEK293 cells were transfected with HA tagged zDHHC isoforms. Following 24 h expression of zDHHC-PATs, cells were lysed and solubilised in lysis buffer; 2 mg/mL C12E10 supplemented with protease inhibitors for 30 min at 4 °C. Lysates were first precleared by incubating with equilibrated streptavidin-beads, then interacting zDHHC-PATs with NCX1 were pulled down using biotinylated NCX1 peptide (Alta Biosciences); corresponds to residues between 740 and 756 (NCX1^740–756^), as described elsewhere [14] and analysed by western blot.

### 4.4 Retention using selective hook (RUSH) system

Retention using selective hook (RUSH) system described previously [28] was adapted based on the aim of our investigation. Briefly, our RUSH system consists of two fundamental components; Golgi- (Golgin-84) or ER- (Ii; a type II protein) resident hook and NCX1^266Y^-SBP reporter (Streptavidin Binding Peptide (SBP) fused to full length NCX1 tagged with YFP at 266 position). Golgin-84 or Ii were fused to streptavidin and tagged with the hemagglutinin (HA) tag, and stably expressed in HEK293 derived FT293 cells using Flp-In T-Rex System (Invitrogen). SBP was fused to NCX1^266Y^ using In-Fusion cloning (TakaraBio Inc.) and expressed in either Golgi or ER hook stably expressing cells.

### 4.5 Confocal microscopy and image analysis

Confocal imaging was performed on HEK293 cells, Golgi- and ER-hook stable cells. 24−48 h after transfection with the plasmids, cells were fixed in 4% PFA for 10 min at room temperature. DNA of the cells were counter-stained with Hoechst to locate the nucleus, where it is indicated. Confocal images were captured with Zeiss LSM880 with Airyscan confocal microscopy. Diode (405−430 nm), Argon (458 nm, 488 nm, 514 nm) and Hene594 (594 nm) lasers were used for detecting blue (Hoechst or CFP), green (YFP) and red (DsRed or mcherry). Colocalization of NCX1 with Golgi- and ER- markers were analysed using Coloc2 macro in Fiji [44]. To quantify the area where NCX1 is populated, original images were first thresholded then the highlighted areas were calculated and presented as the percentage of total cell-area for each cell.

### 4.6 Statistics

Data are presented as mean ± standard error of the mean (SEM). Quantitative differences between groups were calculated by students’ *t*-test using GraphPad Prism. Information about sample size, means and SEM and p-values can be found in Figure legends.

## Funding

We acknowledge financial support from the 10.13039/501100000274British Heart Foundation: FS/14/68/30988 to WF and NF, SP/16/3/32317 and PG/18/60/33957 to WF and a Centre of Research Excellence award RE/18/6/34217.

## Availability of data and material

Further information and requests for resources and reagents should be directed to and will be fulfilled by the corresponding author, William Fuller.

## Author’s contributions

CG: Conceptualization, Investigation, Formal Analysis, Writing – original draft.

AM: Investigation.

XG: Methodology, Resources.

ZK: Methodology, Investigation.

FP: Investigation.

CWK: Methodology, Resources.

ADR: Methodology, Resources.

NJF: Funding Acquisition.

WF: Conceptualization, Project Administration, Funding Acquisition, Supervision, Writing – review & editing.

## Declaration of Competing Interest

The authors report no declarations of interest.
