# Supplementary figures and images for: Insights into the molecular basis of the palmitoylation and depalmitoylation of NCX1
Source: Cell Calcium. 2021 Jul;97:102408. doi: 10.1016/j.ceca.2021.102408 (PMC8278489; doi:10.1016/j.ceca.2021.102408)

# NCX1 266-765

## GOLGI HOOK

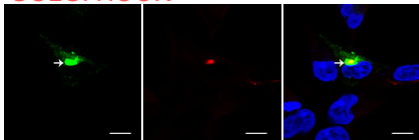

NCX1/HA -Golgin84/Hoechst

## ER HOOK

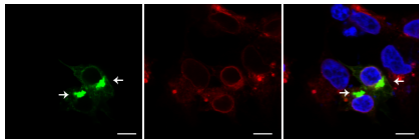

NCX1/HA -li/Hoechst

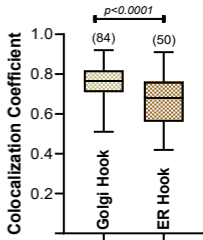

Supplement: Supplementary file 1 [file mmc1.pdf]
