# Supplementary material for: Insights into the molecular basis of the palmitoylation and depalmitoylation of NCX1
Source: Cell Calcium. 2021 Jul;97:102408. doi: 10.1016/j.ceca.2021.102408 (PMC8278489; doi:10.1016/j.ceca.2021.102408)
Supplement: Supplementary file 2 [file mmc2.docx]

**Supplementary Figure: Colocalization of NCX1^266-765^ in Golgi and ER Hook cells**

Confocal images of NCX1^266-765^ in Golgi and ER Hook cells, Scale bar: 10µm (Pearson’s coefficient: 0.75±0.009 (SEM) for NCX1^266-765^/Golgi colocalization (n: 84); 0.67±0.018 (SEM) for NCX1^266-765^/ER colocalization (n: 50); p-value: <0.0001 for NCX1^266-765^/Golgi vs NCX1^266-765^/ER).
